# Relationship between the albumin-corrected anion gap and short-term prognosis among patients with cardiogenic shock: a retrospective analysis of the MIMIC-IV and eICU databases

**DOI:** 10.1136/bmjopen-2023-081597

**Published:** 2024-10-02

**Authors:** Yuxing Wang, Yuhang Tao, Ming Yuan, Pengcheng Yu, Kai Zhang, Hangying Ying, Ruhong Jiang

**Affiliations:** 1 Cardiology, Zhejiang University School of Medicine Sir Run Run Shaw Hospital, Hangzhou, Zhejiang, China

**Keywords:** CARDIOLOGY, INTENSIVE & CRITICAL CARE, Cardiovascular Disease

## Abstract

**Abstract:**

**Objectives:**

We aimed to investigate the association between the albumin-corrected anion gap (ACAG) and the prognosis of cardiogenic shock (CS).

**Design:**

A multicentre retrospective cohort study.

**Setting:**

Data were collected from the Medical Information Mart for Intensive Care (MIMIC-IV) and eICU Collaborative Research Database (eICU-CRD) datasets.

**Participants:**

808 and 700 individuals from the MIMIC-IV and eICU-CRD, respectively, who were diagnosed with CS.

**Primary and secondary outcomes:**

The primary endpoint was short-term all-cause mortality, including intensive care unit (ICU), in-hospital and 28-day mortality. The secondary endpoints were the 28-day free from the ICU duration and the length of ICU stay.

**Results:**

CS patients were divided into two groups according to the admission ACAG value: the normal ACAG group (≤20 mmol/L) and the high ACAG group (> 20 mmol/L). CS patients with higher ACAG values exhibited increased short-term all-cause mortality rates, including ICU mortality (MIMIC-IV cohort: adjusted HR: 1.43, 95% CI=1.05–1.93, p=0.022; eICU-CRD cohort: adjusted HR: 1.38, 95% CI=1.02–1.86, p=0.036), in-hospital mortality (MIMIC-IV cohort: adjusted HR: 1.31, 95% CI=1.01–1.71, p=0.03; eICU-CRD cohort: adjusted HR: 1.47, 95% CI=1.12–1.94, p=0.006) and 28-day mortality (adjusted HR: 1.42, 95% CI: 1.11 to 1.83, p=0.007). A positive linear correlation was observed between the ACAG value and short-term mortality rates via restricted cubic splines. Compared with the AG, the ACAG presented a larger area under the curve for short-term mortality prediction. In addition, the duration of intensive care was longer, whereas the 28-day free from the ICU duration was shorter in patients with a higher ACAG value in both cohorts.

**Conclusion:**

The ACAG value was independently and strongly associated with the prognosis of patients with CS, indicating that the ACAG value is superior to the conventional AG value.

**STRENGTHS AND LIMITATIONS OF THIS STUDY**

- The included patients were from two distinct high-quality datasets with mixed aetiologies of cardiogenic shock (CS).
- We employed restricted cubic splines to reveal the association between the albumin-corrected anion gap value and short-term mortality in CS patients.
- Given its retrospective nature, selection bias cannot be avoided, and detailed information about cardiac function is not available.

## Introduction

 Cardiogenic shock (CS), a life-threatening clinical condition, is characterised by acute end-organ hypoperfusion resulting from reduced cardiac output.^1^ Despite substantial progress achieved in CS management over the past three decades, the mortality rate of CS remains unexpectedly high, making it a formidable challenge within the intensive care unit (ICU).^2^ Notably, the 1 year mortality rate of CS patients is approximately 50%–60%, with a substantial portion of cases (70% to 80%) occurring within the initial 30 to 60 days.^3^ Therefore, early identification of CS patients with a poor prognosis holds paramount clinical importance for tailoring effective risk reduction strategies.

The anion gap (AG), a biomarker reflecting unmeasured anions, is calculated via the following formula: AG (mmol/l) = (sodium+potassium) − (chloride+bicarbonate).^4^ It is extensively used to assess acid‒base disorders and evaluate the prognosis of various diseases in clinical practice.^5^ Nevertheless, the accuracy of the AG in predicting the prognosis of patients in the ICU remains debatable. While some studies have suggested that the AG can effectively predict short-term mortality in patients with critical illness, others have yielded inconclusive results.^6^ In 1985, Gabow reported that the AG value could be influenced by serum albumin levels.^7^ Given that albumin has a negative charge, any fluctuations in albumin levels can impact the final AG measurement.^8^ Consequently, for patients with critical illness in the ICU, the AG may sometimes appear to be pseudonormal since hypoalbuminaemia is very common in the setting of intensive care.^9^ To address this problem, Figge J *et al* introduced the concept of the albumin-corrected anion gap (ACAG) in 1998.^10^ Hatherill *et al* discovered that the ACAG exhibited superior predictive capabilities for metabolic acidosis than did the AG in paediatric patients with shock.^11^ Furthermore, numerous studies have demonstrated the association between the ACAG and the prognosis of critical conditions, including cardiac arrest,^12^ acute myocardial infarction,^13^ acute kidney injury,^14^ sepsis^15^ and acute pancreatitis.^16^

However, to the best of our knowledge, the relationship between the ACAG and the prognosis of CS patients has not been investigated. Furthermore, it remains uncertain whether the ACAG offers an improved ability to predict short-term mortality compared with the AG. Therefore, in this study, our objectives are as follows: (1) to examine the correlation between the ACAG and short-term mortality in patients with CS and (2) to compare the admission values of the AG and ACAG for predicting CS mortality and assessing severity.

## Materials and methods

### Datasets and ethics

In this study, we used the following two publicly accessible datasets: (1) the Medical Information Mart for Intensive Care IV/MIMIC-IV v2.2 dataset (2008–2019)^17^ and (2) the eICU Collaborative Research Database/eICU-CRD dataset (2014–2015).^18^ The MIMIC-IV is an updated version of the MIMIC-III, containing depersonalised data of 73 181 ICU stays for 50 906 unique patients at the Beth Israel Deaconess Medical Centre between 2008 and 2019 (a single-centre dataset). The eICU-CRD is also a deidentified database and contains 200 859 ICU stays for 139 367 unique patients admitted to 335 ICUs at 208 hospitals across the USA (a multicentre dataset). Importantly, as there is no shared hospital involvement between the MIMIC and eICU datasets, the eICU-CRD dataset remains entirely independent of the MIMIC-IV dataset.

The first author successfully completed the online course and passed the Examination for Protecting Human Research Participants (Record ID: 11841860). Hence, he was granted permission to extract data from the two datasets mentioned above. Given that all identifying information had been removed, our study was considered exempt from ethical review by the institutional research board. Patients or the public were not involved in the design, conduct, reporting or dissemination plans of our research.

### Patient and public involvement

Neither the patients nor the members of the public were involved in any part of this study.

### Study population and endpoints

This was a multicentre, retrospective, observational study. CS was defined on the basis of the diagnostic codes from the MIMIC-IV and eICU-CRD databases. These codes are in accordance with standard clinical definitions. We excluded those who were younger than 18 years old, had a length of stay (LOS) in the ICU or hospital of less than 24 hours or lacked AG values or albumin levels within the first 24 hours of ICU admission. For patients with multiple ICU admissions, we included only the first ICU stay for analysis. The AG value was calculated via the following formula: AG (mmol/l) = (sodium+potassium) − (chloride+bicarbonate). The ACAG value was determined as follows: ACAG (mmol/l) = [4.4-{albumin(g/dl)}] *2.5+AG.^11^ Additionally, we categorised the enrolled patients into two groups according to the ACAG admission value and previous studies^14 15^ : the normal ACAG group (<20 mmol/L) and the high ACAG group (≥20 mmol/L).

The primary endpoint of this study was short-term all-cause mortality, which included ICU mortality, in-hospital mortality and 28-day mortality (not available in the eICU-CRD dataset). The secondary endpoints included 28-day free from the ICU duration (not available in the eICU-CRD dataset) and LOS in the ICU. The 28-day free from the ICU duration is a composite outcome that integrates both mortality and LOS in the ICU. It was calculated as 28 minus the days spent in the ICU during the first 28 days, and the dead patients were assigned a value of zero. The LOS in the ICU was defined as the duration that intensive care was required and was calculated based on the time to discharge alive from the ICU, with death in the ICU as a competing risk.

### Variable extraction

We extracted the variables with structured query language in Navicat Premium (version 15.0.12). The codes for data extraction were based on https://github.com/MIT-LCP/mimic-code and https://github.com/MIT-LCP/eicu-code. For each patient, we collected a wide range of variables, including demographic information, comorbidities, Sequential Organ Failure Assessment (SOFA) score, vital signs and laboratory data. Demographic information included age at admission, sex, weight/body mass index and race. Acute myocardial infarction, hypertension, atrial fibrillation, valvular disease, cardiomyopathy, acute kidney injury/acute renal failure, chronic obstructive pulmonary disease, diabetes and malignancy were identified as comorbidities. Vital signs included heart rate, respiratory rate, systolic blood pressure, diastolic blood pressure, mean blood pressure and oxygen saturation. Additionally, we collected laboratory data, which included white blood cell count, haemoglobin, platelet, bilirubin, creatinine, sodium, potassium, chloride, bicarbonate and albumin levels, and AG and ACAG values.

All vital signs, laboratory data and SOFA scores were extracted and calculated within the first 24 hours of ICU admission. If a variable was measured multiple times within the initial 24 hours of ICU admission, we used the first recorded value for analysis.

### Statistical analysis

To address missing values, we initially conducted multiple imputation using chained equations. In the MIMIC-IV cohort, the percentage of incomplete cases was 3.1%, and in the eICU-CRD cohort, it was 16.7%. Accordingly, we generated five datasets for MIMIC-IV and 17 datasets for eICU-CRD for further analysis, and the results were combined according to Rubin’s rules.^19^

We compared the baseline characteristics of the enrolled patients on the basis of their hospital survival status and ACAG value. Categorical variables are presented as numbers plus percentages and were compared via Pearson’s X^2^ test. Shapiro‒Wilk tests were performed to assess the distribution of continuous variables. Since all the continuous variables in the two cohorts were skewed, they are expressed as medians (IQRs) and were compared via the Wilcoxon rank sum test.

Pearson correlation analyses were used to investigate the associations between the AG/ACAG values and the SOFA score. The ability of the AG and ACAG values to predict short-term mortality was compared by the area under the curve (AUC) of the receiver operating characteristic (ROC) curve. A Z test was used to compare the predictive ability of the AG and ACAG values following the methods of Delong *et al*.^20^ Threshold values were determined by identifying the values that provided the highest specificity and sensitivity via the calculation of the Youden index.

To evaluate the relationships between the ACAG value and ICU, in-hospital and 28-day all-cause mortality, the ACAG value was initially analysed as a categorical variable (normal ACAG group and high ACAG group) and then as a continuous variable (ACAG values). Kaplan‒Meier survival curves and Cox proportional hazards regression models were used to calculate the hazard ratios (HRs) and 95% CIs. Furthermore, we investigated the association between the ACAG value and short-term mortality via restricted cubic splines with four knots at 25%, 50%, 75% and 95%. On the basis of previous studies and theoretical considerations, we selected clinically relevant confounding factors as covariates in the regression model. The variance inflation factor was used to test the multicollinearity between each covariate, and the covariates with a high degree of collinearity (variance inflation factor >5) were removed from the regression model. Finally, we constructed two models for adjustments. In Model I, we adjusted for confounders, including age, sex, race and weight/body mass index. In Model II, we further adjusted for acute myocardial infarction, cardiomyopathy, atrial fibrillation, valvular heart disease, diabetes, chronic obstructive pulmonary disease, acute kidney injury, SOFA score, mean blood pressure, oxygen saturation and potassium, chloride, creatinine and total bilirubin levels.

Since ICU death resulted in a shorter LOS, the correlation between the ACAG value and LOS in the ICU was analysed via the Fine‒Grey competing risk model. In this model, a higher HR for earlier alive ICU discharge indicated a shorter LOS, whereas a lower HR indicated a longer LOS in the ICU.

Subgroup analyses were conducted to evaluate the relationships between the ACAG value and 28-day all-cause mortality within various subpopulations, including age (<65 years, ≥65 years), sex (male, female), acute myocardial infarction, atrial fibrillation, valvular disorders, cardiomyopathy, chronic obstructive pulmonary disease, diabetes mellitus, acute kidney injury/acute renal failure, hypoalbuminaemia (<3.5 g/dL, ≥3.5 g/dL) and SOFA score (<8, ≥8), via stratified multivariable Cox proportional hazards model.

All the statistical analyses were performed with R version 4.1.2. A two-sided P value <0.05 was considered statistically significant.

## Results

### Baseline characteristics of the enroled patients

**Figure 1:**
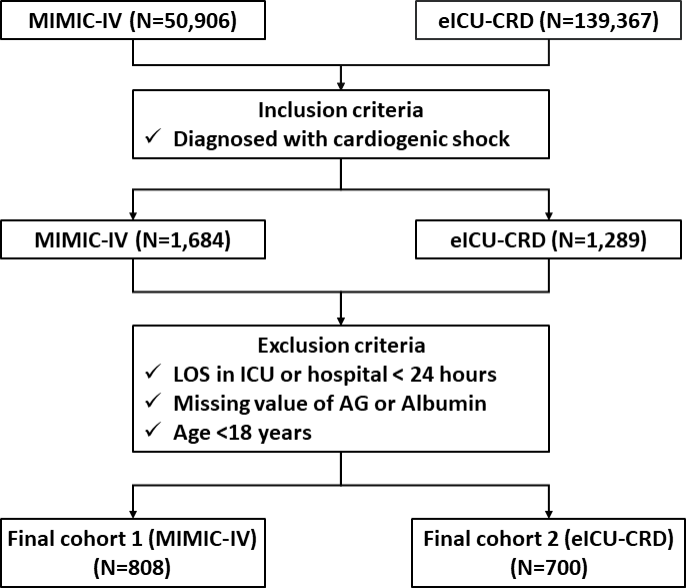
Flow chart of this study. LOS, length of stay; ICU, intensive care unit,; AG, anion gap.

The flowchart of our study is presented in figure 1. The differences between the included and excluded patients are summarised in Online supplemental etable 1. Overall, a total of 808 and 700 individuals diagnosed with CS were enrolled from the MIMIC-IV dataset and eICU-CRD dataset, respectively. The short-term mortality rates of CS patients were similar in both cohorts. Specifically, the ICU mortality rates were 29% and 30%, whereas the in-hospital mortality rates were 36% and 37% in the MIMIC-IV cohort and eICU-CRD cohort, respectively. In the MIMIC-IV cohort, the 28-day all-cause mortality rate was 39%.

Table 1 summarises the baseline characteristics of the enroled patients stratified according to the ACAG value. Patients with a higher ACAG value clearly exhibited a greater predisposition to acute kidney injury/acute renal failure and had elevated SOFA scores; white blood cell counts; and sodium, potassium, creatinine and total bilirubin levels. Compared with those in the normal ACAG group, the short-term mortality rates (including ICU mortality, in-hospital mortality and 28-day mortality) were significantly greater, whereas the 28-day mortality rates in patients in the ICU were notably lower (20^2^,^25^ vs 9 [0–23], p<0.001) in the high ACAG group.

**Table 1:** Baseline characteristics of the enroled patients stratified by the ACAG value in the two cohorts

	MIMIC-IV cohort (n=808)				eICU-CRD cohort (n=700)			
	Overall(n=808)	Normal ACAG (n=416)	Higher ACAG (n=392)	p value	Overall(n=700)	Normal ACAG (n=353)	Higher ACAG (n=347)	p value
Demographic characteristics								
Age	70 (60, 80)	71 (61, 81)	70 (60, 79)	0.3	67 (57, 76)	68 (57, 77)	67 (57, 75)	0.2
Sex				0.2				
Female	347 (43%)	188 (45%)	159 (41%)		255 (36%)	130 (37%)	125 (36%)	
Male	461 (57%)	228 (55%)	233 (59%)		445 (64%)	223 (63%)	222 (64%)	
Weight/BMI*	80 (68, 95)	79 (67, 94)	81 (68, 97)	0.2	28 (24, 33)	28 (24, 33)	28 (24, 33)	0.6
Ethnicity				0.3				0.016*
White	497 (62%)	255 (61%)	242 (62%)		531 (76%)	276 (78%)	255 (73%)	
Black	71 (8.8%)	34 (8.2%)	37 (9.4%)		83 (12%)	32 (9.1%)	51 (15%)	
Hispanic	19 (2.4%)	12 (2.9%)	7 (1.8%)		32 (4.6%)	14 (4.0%)	18 (5.2%)	
Asian	18 (2.2%)	13 (3.1%)	5 (1.3%)		19 (2.7%)	15 (4.2%)	4 (1.2%)	
Others/unknown	203 (25%)	102 (25%)	101 (26%)		35 (5.0%)	16 (4.5%)	19 (5.5%)	
Comorbidities								
AMI	349 (43%)	187 (45%)	162 (41%)	0.3	270 (39%)	152 (43%)	118 (34%)	0.014*
Hypertension	241 (30%)	143 (34%)	98 (25%)	0.004*	365 (52%)	186 (53%)	179 (52%)	0.8
Cardiomyopathy	206 (25%)	105 (25%)	101 (26%)	0.9	119 (17%)	69 (20%)	50 (14%)	0.070
Atrial fibrillation	393 (49%)	199 (48%)	194 (49%)	0.6	144 (21%)	77 (22%)	67 (19%)	0.4
VHD	293 (36%)	150 (36%)	143 (36%)	>0.9	99 (14%)	64 (18%)	35 (10%)	0.002*
AKI/ARF*	573 (71%)	259 (62%)	314 (80%)	<0.001*	323 (46%)	150 (42%)	173 (50%)	0.051
COPD	71 (8.8%)	41 (9.9%)	30 (7.7%)	0.3	101 (14%)	53 (15%)	48 (14%)	0.7
Diabetes	283 (35%)	116 (28%)	167 (43%)	<0.001*	158 (23%)	70 (20%)	88 (25%)	0.080
Malignancy	80 (9.9%)	38 (9.1%)	42 (11%)	0.5	16 (2.3%)	6 (1.7%)	10 (2.9%)	0.3
SOFA	8 (5, 11)	7 (4, 10)	9 (6, 12)	<0.001*	8 (6, 11)	7 (5, 10)	9 (7, 12)	<0.001*
Vital signs								
Heart rate	90 (77, 108)	87 (74, 102)	93 (80, 111)	<0.001*	91 (78, 108)	90 (77, 105)	93 (78, 111)	0.088
Respiratory rate	20 (17, 24)	20 (16, 23)	21 (17, 26)	<0.001*	20 (17, 25)	19 (16, 24)	20 (17, 25)	0.083
Systolic BP	111 (97, 129)	114 (99, 127)	109 (95, 129)	0.2	107 (91, 122)	107 (92, 121)	107 (90, 126)	0.8
Diastolic BP	66 (54, 79)	66 (54, 79)	66 (54, 78)	0.5	62 (50, 75)	62 (50, 73)	62 (50, 77)	0.3
Mean BP	79 (68, 91)	79 (69, 91)	78 (66, 91)	0.4	77 (65, 89)	76 (67, 88)	78 (64, 91)	0.4
SpO_2_	97 (94, 100)	98 (94, 100)	97(93, 100)	0.12	97 (93, 100)	97 (94, 100)	98 (93, 100)	>0.9
Laboratory data								
White blood cell	13 (9, 17)	12 (9, 17)	13 (9, 18)	0.001*	12 (9, 18)	12 (9, 16)	13 (9, 20)	0.002*
Haemoglobin	11.5(9.8, 13.4)	11.7(10.1, 13.5)	11.4(9.6, 13.1)	0.045*	12.1(10.1, 13.9)	12.2(10.3, 14.0)	11.8(9.8, 13.7)	0.2
Platelet	211(152, 278)	210(154, 278)	213(149, 278)	0.8	196(145, 260)	203(151, 253)	192(139, 268)	0.4
Sodium	138(134, 141)	138(135, 141)	137(133, 141)	0.2	137(134, 141)	137(135, 140)	138(133, 141)	>0.9
Potassium	4.4(3.9, 5.0)	4.3(3.8, 4.7)	4.6(3.9, 5.1)	<0.001*	4.2(3.7, 4.9)	4.1(3.7, 4.7)	4.4(3.7, 5.2)	<0.001*
Chloride	103(98, 107)	104 (100,108)	101(96, 106)	<0.001*	103(98, 107)	104(100, 108)	101(96, 105)	<0.001*
Bicarbonate	20 (17, 23)	22 (20, 25)	18 (15, 21)	<0.001*	22 (18, 25)	24 (21, 27)	19 (16, 22)	<0.001*
AG	13 (9, 17)	12 (9, 17)	13 (9, 18)	0.001*	167 (13, 21)	13 (12, 15)	21 (18, 24)	<0.001*
Albumin	3.3 (2.9, 3.7)	3.4 (3.0, 3.7)	3.2 (2.7, 3.6)	<0.001*	3.0 (2.6, 3.5)	3.1 (2.8, 3.6)	2.9 (2.5, 3.4)	<0.001*
ACAG	20.0(17.0, 23.5)	17.1(15.3, 18.5)	23.5(21.8, 26.5)	<0.001*	19.9(16.7, 24.2)	16.7(14.8, 18.3)	24.2(21.9, 28.0)	<0.001*
Creatine	1.4 (1.0, 2.3)	1.2 (0.9, 1.7)	1.8 (1.3, 2.9)	<0.001*	1.5 (1.1, 2.4)	1.3 (0.9, 1.8)	1.8 (1.3, 2.8)	<0.001*
Bilirubin	0.7 (0.4, 1.3)	0.7 (0.4, 1.0)	0.8 (0.5, 1.5)	<0.001*	0.8 (0.5, 1.4)	0.8 (0.5, 1.3)	0.9 (0.5, 1.6)	0.055
Outcomes								
LOS in the ICU	5 (3, 9)	5 (3, 9)	5 (3, 9)	0.5	5 (3, 9)	5 (3, 8)	5 (3, 9)	0.3
LOS in hospital	10 (5, 17)	10 (6, 17)	10 (5, 18)	0.3	8 (5, 14)	9 (5, 15)	8 (4, 14)	0.017*
ICU death	231 (29%)	85 (20%)	146 (37%)	<0.001*	211 (30%)	87 (25%)	124 (36%)	0.001*
Hospital death	289 (36%)	122 (29%)	167 (43%)	<0.001*	260 (37%)	102 (29%)	158 (46%)	<0.001*
28-day death†	315 (39%)	126 (30%)	189 (48%)	<0.001*				
28-day free from the ICU duration†	17 (0, 24)	20 (2, 25)	9 (0, 23)	<0.001*				

pp<0.05*

* Body weight and acute kidney injury are shown for the MIMIC-IV cohort, whereas body mass index and acute renal failure are presented for the eICU-CRD cohort because of data availability

† 28-day all-cause mortality and 28-day free from the ICU duration were reported for the MIMIC-IV cohort.

BMI, body mass index; AMI, acute myocardial infarction; AKI, acute kidney injury; ARF, acute renal failure; COPD, chronic obstructive pulmonary disease; SOFA, sequential organ failure assessment; BP, blood pressure; AG, anion gap; ACAG, albumin-corrected anion gap; LOS, length of stay; ICU, intensive care unit

Furthermore, the baseline characteristics of the enroled patients stratified according to hospital survival status are summarised in online supplemental etable 2. Notably, we found that the ACAG value was significantly greater in the group of patients who did not survive in the hospital, both in the MIMIC-IV cohort (21.0 [18.0–25.3] vs 19.0 [16.5–22.5], p<0.001) and in the eICU-CRD cohort (22.0 [17.7–27.0] vs 19.0 [16.2–23.0], p<0.001). Additionally, among the nonsurvivors during hospitalisation, we observed a higher rate of acute kidney injury/acute renal failure; lower haemoglobin, albumin and bicarbonate levels; and higher age, creatinine levels and SOFA scores.

### Comparison of the AG and ACAG values for mortality prediction and severity assessment

The predictive performance of the ACAG value versus the AG value for ICU, in-hospital and 28-day all-cause mortality was assessed through ROC curve analysis (e*Fig. 1*). As shown in table 2, the ACAG value outperformed the AG value for short-term mortality prediction, including ICU mortality (MIMIC-IV cohort: AUC: 0.654 [95% CI: 0.613 to 0.696] vs 0.632 [95% CI: 0.589 to 0.674], Z=2.99, p=0.003; eICU-CRD cohort: AUC: 0.613 [95% CI: 0.566 to 0.660] vs 0.594 [95% CI: 0.546 to 0.642], Z=2.99, p=0.003), in-hospital mortality (MIMIC-IV cohort: ACU: 0.629 [95% CI: 0.589 to 0.669] vs 0.599 [95% CI: 0.558 to 0.641], Z=4.13, p<0.001; eICU-CRD cohort: AUC: 0.628 [95% CI: 0.585 to 0.671] vs 0.603 [95% CI: 0.559 to 0.647], Z=3.92, p<0.001), and 28-day mortality prediction (MIMIC-IV cohort: AUC: 0.641 [95% CI: 0.602 to 0.680] vs 0.614 [95% CI: 0.574 to 0.654], Z=3.95, p<0.001).

**Table 2:** ROC curve analysis of AG/ACAG values and short-term mortality

	Factor	AUC	95% CI	Cut-off	Sensitivity	Specificity	Youden’s index
ICU mortality(MIMIC-IV)	AG	0.654	0.613 to 0.696	15.5	0.758	0.426	0.184
	ACAG	0.632	0.589 to 0.674	19.6	0.680	0.532	0.212
ICU mortality(eICU-CRD)	AG	0.594	0.546 to 0.642	18.1	0.526	0.654	0.180
	ACAG	0.613	0.566 to 0.660	25.4	0.351	0.857	0.208
Hospital mortality(MIMIC-IV)	AG	0.599	0.558 to 0.641	20.5	0.346	0.796	0.142
	ACAG	0.629	0.589 to 0.669	24.6	0.322	0.869	0.191
Hospital mortality(eICU-CRD)	AG	0.603	0.559 to 0.647	18.1	0.523	0.673	0.196
	ACAG	0.628	0.585 to 0.671	21.6	0.527	0.705	0.232
28-day mortality(MIMIC-IV))	AG	0.614	0.574 to 0.654	21.5	0.295	0.870	0.165
	ACAG	0.641	0.602 to 0.680	22.9	0.400	0.805	0.205

AUC, area under curve; CI, confidence interval; AG, anion gap; ACAG, albumin-corrected anion gap

Additionally, we conducted correlation analyses to investigate the association between the AG/ACAG values and the SOFA score via Pearson’s method. As depicted in online supplemental efigure 2, in both cohorts, we observed positive correlations between the AG and ACAG values and the SOFA score (both p values<0.001). Intriguingly, we found that the correlation coefficient for the ACAG value was significantly greater than that for the AG value (MIMIC-IV cohort: AG: R=0.28 vs. ACAG: R=0.35; eICU-CRD cohort: AG: R=0.30 vs. ACAG: R=0.35). These findings highlight the strong positive correlation between the ACAG value and the SOFA score, underscoring its potential as a valuable prognostic indicator.

### Increased ACAG value is correlated with increased risk of short-term morality

As shown in *eFig. 3*, the Kaplan–Meier survival curve revealed an increased 28-day all-cause mortality rate among patients with a higher ACAG value (HR: 1.85, 95% CI: 1.48 to 2.32, log-rank test, p value <0.001) in the MIMIC-IV cohort. Furthermore, even after adjusting for confounding variables in Model II, we observed that the individuals whose ACAG value was evaluated still presented an increased 28-day all-cause mortality rate (adjusted HR: 1.42, 95% CI: 1.11 to 1.83, p=0.007).

Similarly, the relationship between the ACAG value and ICU/in-hospital mortality was also assessed through multivariable Cox regression models. As presented in table 3, in comparison with the normal ACAG group, the high ACAG group experienced increased rates of ICU mortality (MIMIC-IV cohort: 1.43, 95% CI=1.05–1.93, p=0.022; eICU-CRD cohort: adjusted HR: 1.38, 95% CI=1.02–1.86, p=0.036) and in-hospital mortality (MIMIC-IV cohort: adjusted HR: 1.31, 95% CI=1.01–1.71, p=0.03; eICU-CRD cohort: adjusted HR: 1.47, 95% CI=1.12–1.94, p=0.006).

**Table 3:** Association between the ACAG value and short-term all-cause mortality

	Crude model		Model I		Model II	
	HR (95% CI)	p value	HR (95% CI)	p value	HR (95% CI)	p value
28-day mortality (MIMIC-IV cohort)						
ACAG (per one unit)	1.07 (1.06 to 1.09)	<0.001	1.08 (1.06 to 1.10)	<0.001	1.05 (1.03 to 1.07)	<0.001
Higher ACAG level	1.85 (1.48 to 2.32)	<0.001	1.90 (1.52 to 2.39)	<0.001	1.42 (1.11 to 1.83)	0.007
ICU mortality (MIMIC-IV cohort)						
ACAG (per one unit)	1.06 (1.04 to 1.09)	<0.001	1.07 (1.05 to 1.09)	<0.001	1.04 (1.01 to 1.06)	0.005
Higher ACAG level	1.74 (1.33 to 2.28)	<0.001	1.87 (1.43 to 1.91)	<0.001	1.43 (1.05 to 1.93)	0.022
ICU mortality (eICU-CRD cohort)						
ACAG (per one unit)	1.06 (1.04 to 1.08)	<0.001	1.07 (1.05 to 1.09)	<0.001	1.06 (1.03 to 1.09)	<0.001
Higher ACAG level	1.61 (1.22 to 2.11)	<0.001	1.65 (1.25 to 2.17)	<0.001	1.38 (1.02 to 1.86)	0.036
In-hospital mortality (MIMIC-IV cohort)						
ACAG (per one unit)	1.06 (1.04 to 1.08)	<0.001	1.06 (1.04 to 1.08)	<0.001	1.04 (1.02 to 1.07)	<0.001
Higher ACAG level	1.51 (1.20 to 1.91)	<0.001	1.58 (1.25 to 2.01)	<0.001	1.31 (1.01 to 1.71)	0.041
In-hospital mortality (eICU-CRD cohort)						
ACAG (per one unit)	1.06 (1.04 to 1.08)	<0.001	1.07 (1.05 to 1.09)	<0.001	1.05 (1.02 to 1.07)	<0.001
Higher ACAG level	1.81 (1.41 to 2.33)	<0.001	1.86 (1.44 to 2.39)	<0.001	1.47 (1.12 to 1.94)	0.006

Model I was adjusted for age, sex, race, and weight/body mass index.

Model II was adjusted for age, sex, race, weight/body mass index, acute myocardial infarction, cardiomyopathy, atrial fibrillation, valvular heart disease, diabetes, chronic obstructive pulmonary disease, acute kidney injury, SOFA score, mean blood pressure, oxygen saturation, potassium, chloride, creatine, and total bilirubin.

ACAG, albumin-corrected anion gap; HR, hazard ratio; CI, confidence interval

### Linear relationship between the ACAG value and short-term all-cause mortality

We extended our analysis to assess the association between the ACAG value and short-term all-cause mortality rates. As presented in table 3, the adjusted HRs with 95% CIs were 1.05 (1.03 to 1.07) for 28-day mortality, 1.04 (1.01 to 1.06) for ICU mortality and 1.04 (1.02 to 1.07) for in-hospital mortality in the MIMIC-IV cohort, and 1.06 (1.03 to 1.09) for ICU mortality and 1.05 (1.02 to 1.07) for in-hospital mortality in the eICU-CRD cohort.

**Figure 2:**
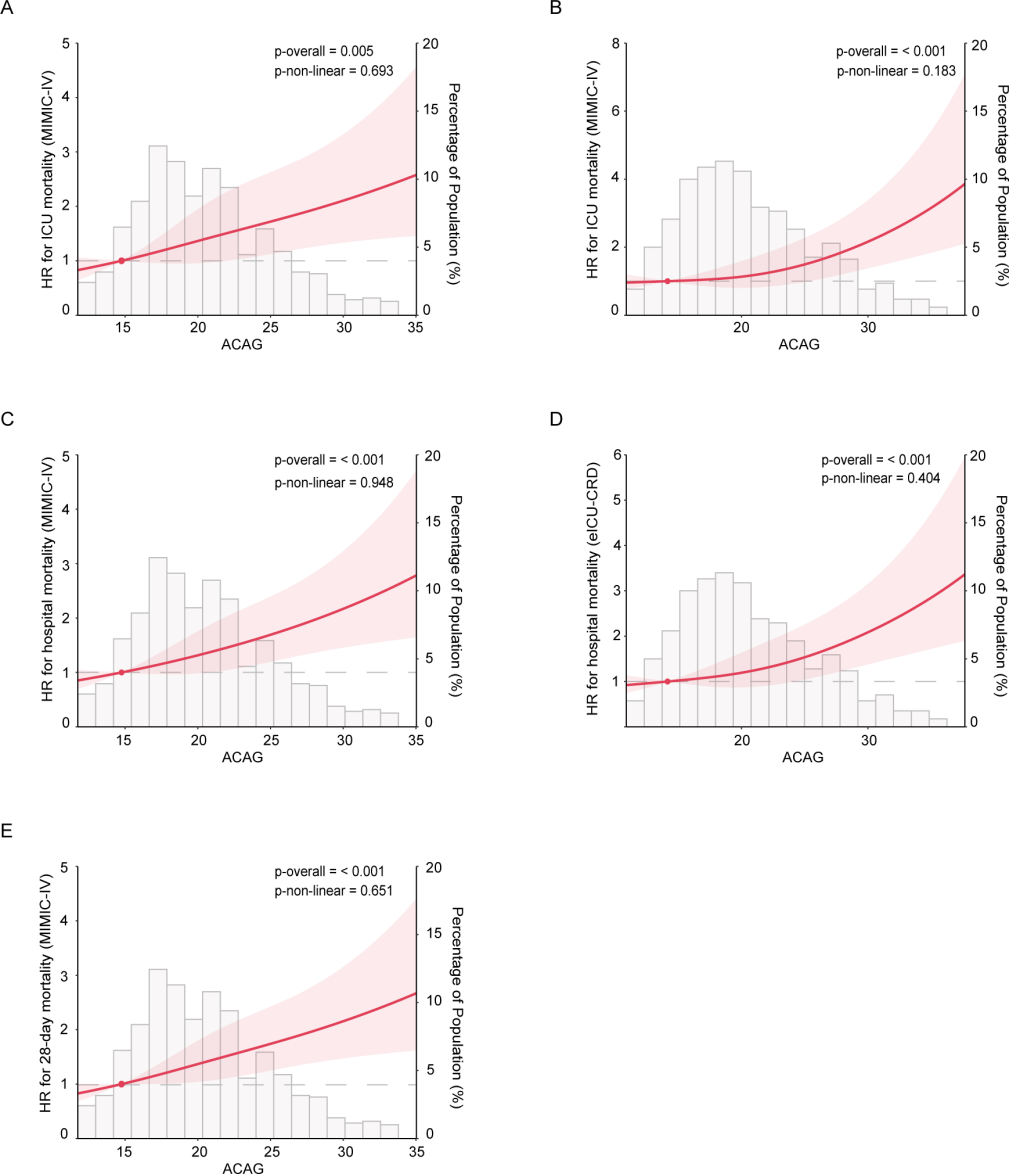
Restricted cubic spline for the associations between the ACAG value and short-term mortality. (A) and (B) The ICU mortality rates. (C) and (D) In-hospital mortality rates in the MIMIC-IV and eICU-CRD cohorts, respectively. (E) The 28-day mortality data. The solid lines represent the adjusted hazard ratios (aHRs) and 95% CIs after multivariable adjustment in Model II. Histograms represent the distribution of the ACAG value in the two cohorts. HR, hazard ratio; ICU, intensive care unit; ACAG, albumin-corrected anion gap.

To further investigate the relationship between the ACAG value and short-term all-cause mortality rates, we used adjusted restricted cubic splines. As shown in figure 2, we observed a linear correlation between the ACAG value and short-term all-cause mortality, which included 28-day mortality (MIMIC-IV cohort: p for overall <0.001, p for nonlinear=0.651), ICU mortality (MIMIC-IV cohort: p for overall<0.001, p for nonlinear=0.693; eICU-CRD cohort: p for overall<0.001, p for nonlinear=0.183) and in-hospital mortality (MIMIC-IV cohort: p for overall<0.001, p for nonlinear=0.948; eICU-CRD cohort: p for overall<0.001, p for nonlinear=0.404) in both cohorts. These findings suggest that a one-unit increase in the ACAG value is associated with an approximately 5% increase in short-term all-cause mortality rates among patients with CS.

### Association of the ACAG value and earlier alive discharge from the ICU

The cumulative incidence ratio (CIR) of earlier discharge alive from the ICU among the different ACAG value groups is shown in *eFig. 4*. Obviously, the unadjusted CIR for earlier alive discharge from the ICU was significantly greater in the normal ACAG group. The robustness of the results was further confirmed via Fine‒Grey competing risk models after adjusting for confounding variables (online supplemental etable 3). In the MIMIC-IV cohort, the adjusted HR (95% CI) for the relationship between the ACAG value and earlier alive discharge from the ICU was 0.77 (95% CI=0.65 to 0.92; p=0.004). However, in the eICU-CRD cohort, this relationship did not reach statistical significance (adjusted HR: 0.85, 0.69 to 1.04; p=0.140).

Additionally, the ACAG value was analysed as a continuous variable. Intriguingly, the association between the ACAG value and earlier discharge was statistically significant in both cohorts, with adjusted HRs (95% CI) of 0.96 (95% CI=0.94 to 0.98; p<0.001) in the MIMIC-IV cohort and 0.97 (95% CI=0.95 to 0.99; p=0.001) in the eICU-CRD cohort. In summary, the ACAG value was inversely associated with earlier discharge from the ICU for patients with CS.

### Subgroup analysis

Subgroup analysis was conducted to assess the consistency of the association between the ACAG value and 28-day all-cause mortality across various subpopulations, including age groups (<65 years, ≥65 years), sexes (male, female), SOFA scores (<8, ≥8) and different clinical conditions, such as acute myocardial infarction, cardiomyopathy, atrial fibrillation, valvular disorders, chronic obstructive pulmonary disease, diabetes mellitus, acute kidney injury and hypoalbuminaemia (<3.5 g/dL, ≥3.5 g/dL). Adjustments for confounding factors were made as in Model II. As depicted in e*Fig. 5*, all p values for the interaction tests within different subgroups were greater than 0.05, indicating that the relationship between the ACAG value and 28-day all-cause mortality remained stable and consistent across various subpopulations.

## Discussion

In this large-sample retrospective study based on two distinct publicly accessible datasets, we investigated the association of the ACAG value, a novel biomarker indicating metabolic acid load, with the short-term prognosis of CS patients with mixed aetiologies. The main findings of our study are as follows: (1) the ACAG value is strongly and independently associated with short-term all-cause mortality rates (including ICU, in-hospital and 28-day mortality) and the duration of intensive care required in patients with CS, even after adjusting for disease severity via the SOFA score; (2) the ACAG value outperforms the AG value in its ability to predict short-term mortality and evaluate the severity of CS.

It is widely acknowledged that metabolic acidosis is a frequent event in the setting of intensive care and has been consistently demonstrated to be associated with adverse outcomes in individuals with critical illness.^21^ Notably, in patients with severe cardiovascular disorders, particularly those suffering from CS, acidaemia may trigger a detrimental cycle by impairing cardiac contractile function, inducing malignant arrhythmias and exacerbating circulatory failure.^22^ Additionally, severe acidaemia may further compromise the response of the cardiovascular system to catecholamines and weaken the effectiveness of vasopressors to reverse hypotension.^23^ A previous study demonstrated that the severity of acidosis is strongly and positively correlated with both the degree of shock and short-term mortality rates in CS patients.^24^

As one of the simplest methods for assessing acid‒base balance, the AG is a widely used biomarker in clinical practice. The relationship between the AG value and short-term mortality in patients with critical illness has been extensively investigated.^25^ A previous study demonstrated a J-shaped association between the AG value and the 30-day all-cause mortality rate in patients with CS on the basis of the MIMIC-III dataset.^26^ Similarly, our study revealed that the AG value was significantly greater in nonsurvivors than in survivors (MIMIC-IV cohort: 18^15^,^22^ vs 16,^14^,^20^ p<0.001; eICU-CRD cohort: 18^14^,^23^ vs 16,^13^,^19^ p<0.001) in our study. Moreover, the AG value has also been used for risk stratification in the setting of acute cardiovascular care. Recently, a study combined the AG value and SOFA score to create the AG-SOFA score, which displayed improved ability to predict short-term mortality in cardiovascular ICU patients.^27^ Similarly, Eric *et al* incorporated the AG value into the BOS and Melbourne Assessment 2 scores and achieved superior performance over other preexisting risk score systems for CS prognostication.^28^ However, the physiological AG value primarily consists of inorganic phosphate and albuminate, which are weak anions derived from serum albumin.^5^ Given the involvement of albumin in acid‒base equilibrium, the interpretation of acid‒base data may be limited.^29^ Theoretically, hypoalbuminaemia can lead to a decrease in albuminate levels, resulting in a reduction in AG values.^10^ Therefore, in the case of a patient with hypoalbuminaemia and a normal AG value, it might indicate the presence of plasma acids. Similarly, we might underestimate the severity of metabolic acidosis on the basis of the AG value for patients with low albumin levels. Notably, hypoalbuminaemia is very common among patients with critical illnesses and has been demonstrated to be associated with unfavourable outcomes, including higher rates of short-term mortality and a longer LOS in the ICU. The incidence of hypoalbuminaemia is striking in patients with CS, with a reported rate of 75% from the previous CardShock study.^30^ Similarly, our study revealed an exceptionally high frequency of hypoalbuminaemia in patients with CS. Specifically, the incidence of hypoalbuminaemia (defined as an albumin concentration <3.5 g/dL) was 58.4% (472/808) in the MIMIC-IV cohort and 74.1% (519/700) in the eICU-CRD cohort. Furthermore, a recent study established that the serum albumin concentration is an independent predictor of short-term mortality in CS patients.^20^ Similarly, in this study, we found that the albumin level was significantly lower in the hospital death group than in the survival group (MIMIC-IV cohort: 3.4 [3.0–3.7] vs 3.1 [2.7–3.6], p<0.001; eICU-CRD cohort: 3.1 [2.7–3.6] vs 2.9 [2.5–3.3], p<0.001).

The ACAG value, which combines the AG value and serum albumin level, has been proposed as a replacement for the AG value in differentiating acidosis caused by acid load or base deficit from an expert consensus panel in metabolic acidosis management.^31^ As a ubiquitous abnormality in patients with critical illnesses, hypoalbuminaemia has been demonstrated to complicate the interpretation of acid–base data when diagnostic methods based on base excess or plasma bicarbonate concentration are used alongside the AG value.^29^ In the presence of hypoalbuminaemia, taking albumin levels into account can reveal the presence of plasma acid, which might otherwise be overlooked when relying solely on the AG or base excess values. Previous studies have demonstrated that the ACAG value is a superior predictor compared with the conventional AG value for short-term prognosis prediction in patients with critical illnesses such as cardiopulmonary arrest,^12^ acute myocardial infarction^13^ and sepsis.^15^ Therefore, we hypothesised that the ACAG value may perform better than the AG value does, particularly in a population at high risk for metabolic acidosis and hypoalbuminaemia. As previously discussed, patients with CS are not only prone to hypoalbuminaemia but also susceptible to metabolic acidosis. Hence, we posited that the ACAG value might outperform the AG value for risk stratification in the context of CS. In this study, we compared the use of the AG and ACAG values for mortality prediction and severity assessment in CS patients in two cohorts. Through ROC curve analysis, we found that the ACAG value had the highest AUC and Youden’s index for short-term mortality prediction in both cohorts, suggesting that the ACAG value has a better ability to predict short-term mortality than the AG value does for CS. Furthermore, using Spearman’s methods, we discovered that both the AG and ACAG values were positively correlated with the SOFA score. Importantly, the correlation coefficients with the SOFA score were significantly greater for the ACAG value than for the AG value. Taken together, our findings support the superiority of the ACAG value in predicting prognosis and estimating disease severity in patients with CS.

As a medical emergency requiring prompt evaluation and intervention, the mortality risk of CS is highest during the initial 48 hours following the onset of shock.^32^ Therefore, mortality assessment in CS patients should be performed as early as possible after ICU admission. Given the rapid and widespread availability of the AG value and albumin level in clinical practice, we recommend the inclusion of the baseline ACAG level as a prognostic biomarker for patients with CS.

Our study has notable strengths. First, this is a pioneering study to explore the association between the ACAG value and the prognosis of CS. Second, CS patients were from a diverse and heterogeneous patient population with mixed aetiologies, enhancing its relevance and applicability to real-world clinical scenarios. Third, the data in this study are derived from two distinct high-quality datasets, and the results are consistent with each other. However, several limitations of this study deserve discussion. First, owing to the retrospective nature of the study, selection bias cannot be avoided. Second, detailed information about cardiac function (such as left ventricular ejection fraction and ventricular size) and other important cardiac biomarkers (such as troponin and N-terminal pro-brain natriuretic peptide levels) was not included in this study because of the large amount of missing data. Third, we could not calculate the CS stages based on the Society for Cardiovascular Angiography and Interventions guidelines accurately because of specific data limitations in the MIMIC-IV and eICU databases. Fourth, the association between the ACAG value and short-term mortality was established on the basis of the first ACAG value within the first 24 hours of ICU admission. Monitoring dynamic changes in the ACAG value may be valuable for patients with CS. However, further studies are needed to explore the relationship between dynamic changes in the ACAG value and mortality in patients with CS.

## Conclusion

In conclusion, we found that the baseline ACAG value following ICU admission independently predicts short-term mortality in patients with CS, which is better than the AG value. Given the high mortality risk of CS during the early phase of ICU admission, the baseline ACAG value may help clinicians identify patients at high risk of mortality. Therefore, we propose incorporating the baseline ACAG value into risk stratification systems for CS.

## supplementary material

10.1136/bmjopen-2023-081597 online supplemental file 1

## Data Availability

Data are available upon reasonable request.
