# Supplementary material for: Relationship between the albumin-corrected anion gap and short-term prognosis among patients with cardiogenic shock: a retrospective analysis of the MIMIC-IV and eICU databases
Source: BMJ Open. 2024 Oct 2;14(10):e081597. doi: 10.1136/bmjopen-2023-081597 (PMC11448225; doi:10.1136/bmjopen-2023-081597)

Albumin corrected anion gap is associated with the prognosis of cardiogenic shock: a multi-center retrospective study

Online Supplement material

eTable1: Baseline characteristics of included and excluded patients in two cohorts

eTable2: Baseline characteristics of enrolled patients stratified by hospital survival status in two cohorts

eTable3: Association of ACAG and earlier discharge alive in ICU

eFigure1: ROC curve analysis of AG, ACAG and ICU mortality (A: MIMIC-IV cohort; B: eICU-CRD cohort), in-hospital morality (C: MIMIC-IV cohort; D: eICU-CRD cohort), and 28-day mortality (E: MIMIC-IV cohort)

eFigure2: Pearson correlation analyses of AC/ACAG and SOFA score in MIMIC-IV cohort (A, C) and eICU-CRD cohort (B, D)

eFigure3: Kaplan–Meier survival curve of ACAG levels and 28-day all-cause mortality

eFigure4: Cumulative incidence ratio of earlier discharge alive in the ICU in MIMIC-IV cohort (A) and eICU-CRD cohort (B)

eFigure5: Subgroup analysis

eTable1: Baseline characteristics of included and excluded patients in two cohorts

	MIMIC-IV cohort (n=1684)				eICU-CRD cohort (n=1289)			
	Overall (n=1684)	Excluded (n=876)	Included (n=808)	p-value	Overall (n=1289)	Excluded (n=589)	Included (n=700)	p-value
Demographic characteristics								
Age	72 (61, 81)	73 (63, 81)	70 (60, 80)	0.006*	68 (57, 77)	69 (58, 78)	67 (57, 76)	0.2
Gender				0.062				0.8
Female	684 (41%)	337 (38%)	347 (43%)		473 (37%)	218 (37%)	255 (36%)	
Male	1000 (59%)	539 (60%)	461 (57%)		816 (64%)	371 (65%)	445 (62%)	
Weight/BMI[a]	80 (68, 95)	80 (68, 95)	80 (68, 95)	0.5	28 (24, 33)	28 (24, 33)	28 (24, 33)	0.4
Ethnicity				0.3				0.2
White	1072 (64%)	575 (66%)	497 (62%)		987 (77%)	456 (77%)	531 (76%)	
Black	144 (8.6%)	73 (8.3%)	71 (8.8%)		136 (11%)	53 (9.0%)	83 (12%)	
Hispanic	32 (1.9%)	13 (1.5%)	19 (2.4%)		62 (4.8%)	30 (5.1%)	32 (4.6%)	
Asian	40 (2.4%)	22 (2.5%)	18 (2.2%)		29 (2.2%)	10 (1.7%)	19 (2.7%)	
Others/unknown	396 (24%)	193 (22%)	203 (25%)		75 (5.8%)	40 (6.8%)	35 (5.0%)	
Comorbidities								
AMI	719 (43%)	370 (42%)	349 (43%)	0.7	489 (38%)	219 (37%)	270 (39%)	0.6
Hypertension	490 (29%)	249 (28%)	241 (30%)	0.5	666 (52%)	301 (51%)	365 (52%)	0.7
Cardiomyopathy	431 (26%)	225 (26%)	206 (25%)	>0.9	221 (17%)	102 (17%)	119 (17%)	0.9
Atrial fibrillation	856 (51%)	463 (53%)	393 (49%)	0.084	250 (19%)	106 (18%)	144 (21%)	0.2
VHD	660 (39%)	367 (42%)	293 (36%)	0.018*	184 (14%)	85 (14%)	99 (14%)	0.9
AKI/ARF[a]	1,118 (66%)	549 (63%)	569 (70%)	<0.001*	533 (41%)	210 (36%)	323 (46%)	<0.001*
COPD	142 (8.4%)	71 (8.1%)	71 (8.8%)	0.6	191 (14%)	90 (15%)	101 (14%)	0.7
Diabetes	609 (36%)	326 (37%)	283 (35%)	0.4	294 (23%)	136 (23%)	158 (23%)	0.8
Malignancy	146 (8.7%)	66 (7.5%)	80 (9.9%)	0.085	28 (2.2%)	12 (2.0%)	16 (2.3%)	0.8

SOFA	8 (5, 11)	8 (5, 10)	8 (5, 11)	0.003	8 (5, 11)	7 (4, 10)	8 (6, 11)	<0.001*
Vital signs								
Heart rate	89 (77, 105)	88 (77, 103)	90 (77, 108)	0.2	90 (77, 107)	88 (76, 105)	91 (78, 108)	0.2
Respiratory rate	20 (16, 24)	20 (16, 24)	20 (17, 24)	0.056	20 (16, 24)	20 (16, 24)	20 (17, 25)	0.11
Systolic BP	109 (95, 125)	108 (93, 123)	111 (97, 129)	<0.001	106 (90, 122)	104 (88, 122)	107 (91, 122)	0.056
Mean BP	78 (67, 89)	76 (66, 88)	79 (68, 91)	<0.001	76 (64, 88)	74 (64, 87)	77 (65, 89)	0.019*
Diastolic BP	64 (52, 77)	63 (51, 75)	66 (54, 79)	<0.001	60 (49, 74)	59 (49, 71)	62 (50, 75)	0.011*
SpO2	98 (94, 100)	98 (94, 100)	97 (94, 100)	0.6	97 (93, 100)	97 (93, 100)	97 (93, 100)	0.5
Laboratory data								
White blood cell	13 (9, 17)	12 (9, 17)	13 (9, 17)	0.5	12 (9, 18)	12 (9, 17)	12 (9, 18)	0.8
Hemoglobin	11.5 (9.5, 13.2)	11.4 (9.2, 13.2)	11.5 (9.8, 13.4)	0.058	11.8 (9.9, 13.7)	11.4 (9.6, 13.3)	12.1 (10.1, 13.9)	<0.001*
Platelet	201 (147, 269)	196 (142, 257)	211 (152, 278)	0.001	192 (143, 255)	189 (140, 251)	196 (145, 260)	0.3
Sodium	138 (135, 141)	138 (135, 141)	138 (134, 141)	0.015	138 (134, 141)	138 (134, 141)	137 (134, 141)	>0.9
Potassium	4.3 (3.9, 4.9)	4.3 (3.8, 4.8)	4.4 (3.9, 5.0)	0.022	4.2 (3.7, 4.9)	4.2 (3.7, 4.8)	4.2 (3.7, 4.9)	0.5
Chloride	103 (98, 107)	103 (98, 107)	103 (98, 107)	0.3	103 (98, 107)	103 (98, 108)	103 (98, 107)	0.5
Bicarbonate	21 (18, 24)	21 (18, 24)	20 (17, 23)	<0.001	22 (18, 25)	22 (18, 25)	22 (18, 25)	0.7
AG	17 (14, 20)	16 (13, 20)	17 (14, 21)	0.003	16 (13, 21)	16 (13, 20)	17 (13, 21)	0.035*
Albumin	3.3 (2.8, 3.7)	3.2 (2.6, 3.6)	3.3 (2.9, 3.7)	0.014	3.0 (2.6, 3.4)	2.9 (2.4, 3.4)	3.0 (2.6, 3.5)	0.004*
ACAG	20.3 (17.3, 24.0)	23.5 (19.0, 30.5)	20.0 (17.0, 23.5)	<0.001	20 (17, 25)	21 (18, 27)	20 (17, 24)	<0.001*

Creatine	1.4 (1.0, 2.1)	1.4 (1.0, 2.1)	1.4 (1.0, 2.3)	0.10	1.4 (1.0, 2.3)	1.4 (1.0, 2.1)	1.5 (1.1, 2.4)	0.061
Bilirubin	0.7 (0.5, 1.2)	0.7 (0.5, 1.2)	0.7 (0.4, 1.3)	0.6	0.8 (0.5, 1.4)	0.8 (0.5, 1.4)	0.9 (0.6, 1.4)	0.6

Abbreviation: BMI: body mass index, AMI: acute myocardial infarction, AKI: acute kidney injury, ARF: acute renal failure, COPD: chronic obstructive pulmonary disease, SOFA sequential organ failure assessment, BP: blood pressure, AG: anion gap, ACAG albumin corrected anion gap, LOS: length of stay, ICU: intensive care unit

$p<0.05$*

a: body weight and acute kidney injury were shown in MIMIC-IV cohort while body mass index and acute renal failure were presented in eICU-CRD cohort due to data availability.

eTable2: Baseline characteristics of enrolled patients stratified by hospital survival status in two cohorts

	MIMIC-IV cohort (n=808)				eICU-CRD cohort (n=700)			
	Overall (n=808)	Survivors (n=519)	Non-survivors (n=289)	p-value	Overall (n=700)	Survivors (n=440)	Non-survivors (n=260)	p-value
Demographic characteristics								
Age	70 (60, 80)	69 (59, 79)	74 (63, 81)	0.001*	67 (57, 76)	66 (55, 75)	70 (62, 79)	<0.001*
Gender				0.018*				0.4
Female	347 (43%)	207 (40%)	140 (48%)		255 (36%)	155 (35%)	100 (38%)	
Male	461 (57%)	312 (60%)	149 (52%)		445 (64%)	285 (65%)	160 (62%)	
Weight/BMI[a]	80 (68, 95)	80 (69, 95)	80 (66, 95)	0.7	28 (24, 33)	28 (24, 33)	28 (24, 33)	>0.9
Ethnicity				0.002*				>0.9
White	497 (62%)	336 (65%)	161 (56%)		531 (76%)	332 (75%)	199 (77%)	
Black	71 (8.8%)	51 (9.8%)	20 (6.9%)		83 (12%)	52 (12%)	31 (12%)	
Hispanic	19 (2.4%)	12 (2.3%)	7 (2.4%)		32 (4.6%)	20 (4.5%)	12 (4.6%)	
Asian	18 (2.2%)	13 (2.5%)	5 (1.7%)		19 (2.7%)	14 (3.2%)	5 (1.9%)	
Others/unknown	203 (25%)	107 (21%)	96 (33%)		35 (5.0%)	22 (5.0%)	13 (5.0%)	
Comorbidities								
AMI	349 (43%)	231 (45%)	118 (41%)	0.3	270 (39%)	181 (41%)	89 (34%)	0.070
Hypertension	241 (30%)	160 (31%)	81 (28%)	0.4	365 (52%)	226 (51%)	139 (53%)	0.6
Cardiomyopathy	206 (25%)	143 (28%)	63 (22%)	0.072	119 (17%)	80 (18%)	39 (15%)	0.3
Atrial fibrillation	393 (49%)	254 (49%)	139 (48%)	0.8	144 (21%)	88 (20%)	56 (22%)	0.6
VHD	293 (36%)	203 (39%)	90 (31%)	0.024*	99 (14%)	62 (14%)	37 (14%)	>0.9
AKI/ARF[a]	573 (71%)	334 (64%)	239 (83%)	<0.001*	323 (46%)	180 (41%)	143 (55%)	<0.001*
COPD	71 (8.8%)	46 (8.9%)	25 (8.7%)	>0.9	101 (14%)	56 (13%)	45 (17%)	0.10
Diabetes	283 (35%)	171 (33%)	112 (39%)	0.10	158 (23%)	94 (21%)	64 (25%)	0.3
Malignancy	80 (9.9%)	41 (7.9%)	39 (13%)	0.011*	16 (2.3%)	6 (1.4%)	10 (3.8%)	0.034*

SOFA	8 (5, 11)	7 (4, 10)	10 (7, 12)	<0.001*	8 (6, 11)	7 (5, 10)	10 (8, 13)	<0.001*
Vital signs								
Heart rate	90 (77, 108)	89 (75, 105)	92 (78, 111)	0.066	91 (78, 108)	90 (77, 105)	93 (78, 111)	0.088
Respiratory rate	20 (17, 24)	20 (17, 24)	21 (17, 26)	0.023*	20 (17, 25)	19 (16, 24)	20 (17, 25)	0.083
Systolic BP	111 (97, 129)	113 (98, 129)	110 (96, 125)	0.2	107 (91, 122)	107 (92, 121)	107 (90, 126)	0.8
Mean BP	66 (54, 79)	67 (55, 79)	63 (52, 78)	0.022*	62 (50, 75)	62 (50, 73)	62 (50, 77)	0.3
Diastolic BP	79 (68, 91)	80 (69, 93)	78 (66, 90)	0.075	77 (65, 89)	76 (67, 88)	78 (64, 91)	0.4
SpO2	97 (94, 100)	97 (94, 100)	98 (94, 100)	0.4	97 (93, 100)	97 (94, 100)	98 (93, 100)	>0.9
Laboratory data								
White blood cell	13 (9, 17)	12 (9, 17)	13 (9, 19)	0.013*	12 (9, 18)	12 (9, 17)	13 (9, 20)	0.076
Hemoglobin	11.5 (9.8, 13.4)	11.8 (10.0, 13.7)	11.2 (9.4, 12.5)	<0.001*	12.1 (10.1, 13.9)	12.4 (10.4, 14.2)	11.6 (9.7, 13.4)	0.003*
Platelet	211 (152, 278)	216 (155, 282)	198 (146, 274)	0.076	196 (145, 260)	210 (157, 266)	182 (128, 242)	<0.001*
Sodium	138 (134, 141)	138 (134, 140)	138 (134, 141)	0.6	137 (134, 141)	137 (134, 140)	138 (134, 142)	0.13
Potassium	4.4 (3.9, 5.0)	4.4 (3.9, 4.9)	4.4 (3.8, 5.0)	>0.9	4.2 (3.7, 4.9)	4.2 (3.7, 4.8)	4.3 (3.7, 5.1)	0.15
Chloride	103 (98, 107)	103 (98, 107)	103 (98, 107)	0.6	103 (98, 107)	103 (99, 107)	103 (98, 107)	>0.9
Bicarbonate	20 (17, 23)	21 (18, 24)	20 (16, 23)	<0.001*	22 (18, 25)	22 (19, 25)	21 (17, 24)	0.002*
AG	17 (14, 21)	16 (14, 20)	18 (15, 22)	<0.001*	17 (13, 21)	16 (13, 19)	18 (14, 23)	<0.001*
Albumin	3.3 (2.9, 3.7)	3.4 (3.0, 3.7)	3.1 (2.7, 3.6)	<0.001*	3.0 (2.6, 3.5)	3.1 (2.7, 3.6)	2.9 (2.5, 3.3)	<0.001*
ACAG	20.0 (17.0, 23.5)	19.0 (16.5, 22.5)	21.0 (18.0, 25.3)	<0.001*	19.9 (16.7, 24.2)	19.0 (16.2, 23.0)	22.0 (17.7, 27.0)	<0.001*
Creatine	1.4 (1.0, 2.3)	1.4 (1.0, 2.1)	1.6 (1.1, 2.6)	<0.001*	1.5 (1.1, 2.4)	1.4 (1.1, 2.3)	1.6 (1.2, 2.4)	0.021*

Bilirubin	0.7 (0.4, 1.3)	0.7 (0.5, 1.2)	0.7 (0.4, 1.4)	0.4	0.8 (0.5, 1.4)	0.8 (0.5, 1.4)	0.9 (0.6, 1.5)	0.065

Abbreviation: BMI: body mass index, AMI: acute myocardial infarction, AKI: acute kidney injury, ARF: acute renal failure, COPD: chronic obstructive pulmonary disease, SOFA sequential organ failure assessment, BP: blood pressure, AG: anion gap, ACAG albumin corrected anion gap, LOS: length of stay, ICU: intensive care unit

p<0.05*

a: body weight and acute kidney injury were shown in MIMIC-IV cohort while body mass index and acute renal failure were presented in eICU-CRD cohort due to data availability.

eTable3: Association of ACAG and earlier discharge alive in ICU

	Crude Model		Model I		Model II	
	HR (95%CI)	p-value	HR (95%CI)	p-value	HR (95%CI)	p-value
LOS in ICU (MIMIC-IV cohort)						
ACAG	0.94 (0.92-0.95)	<0.001	0.94 (0.92-0.95)	<0.001	0.96 (0.94-0.98)	<0.001
Higher ACAG	0.62 (0.53-0.73)	<0.001	0.61 (0.52-0.72)	<0.001	0.77 (0.65-0.92)	0.004
LOS in ICU (eICU-CRD cohort)						
ACAG	0.96 (0.95-0.98)	<0.001	0.96 (0.94-0.98)	<0.001	0.97 (0.95-0.99)	0.001
Higher ACAG	0.74 (0.62-0.88)	<0.001	0.73 (0.61-0.88)	<0.001	0.85 (0.69-1.04)	0.140

Model I adjusted for age, gender, race, and weight/body mass index

Model II adjusted for age, gender, race, weight/body mass index, acute myocardial infarction, cardiomyopathy, atrial fibrillation, valvular heart disease, diabetes, chronic obstructive pulmonary disease, acute kidney injury, SOFA score, mean blood pressure, oxygen saturation, potassium, chloride, creatinine, and total bilirubin.

eFigure1: ROC curve analysis of AG, ACAG and ICU mortality (A: MIMIC-IV cohort; B: eICU-CRD cohort), in-hospital morality (C: MIMIC-IV cohort; D: eICU-CRD cohort), and 28-day mortality (E: MIMIC-IV cohort)

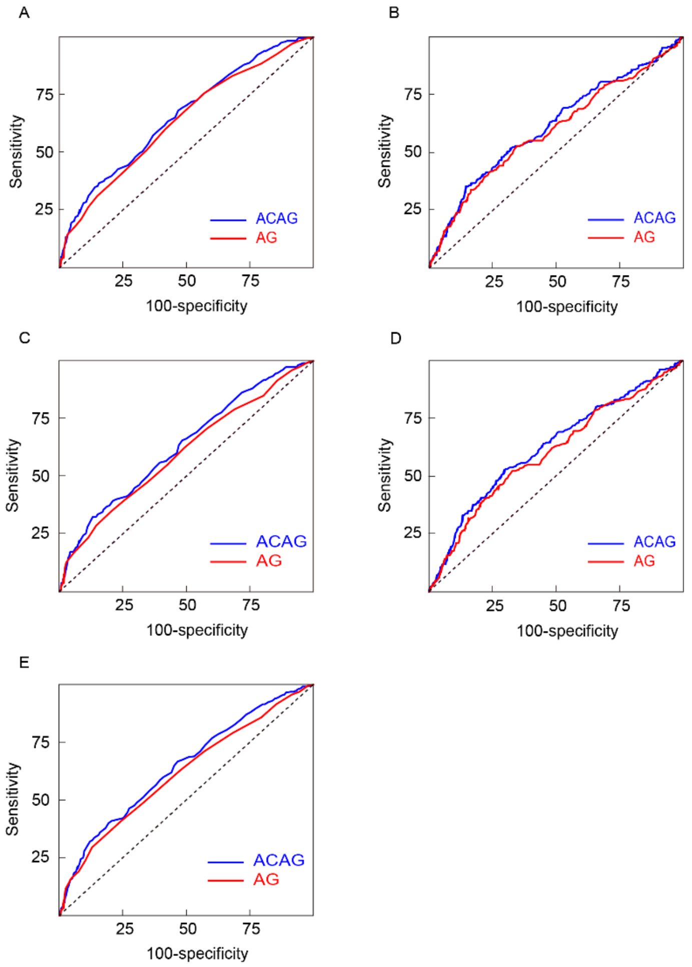

eFigure2: Pearson correlation analyses of AC/ACAG and SOFA score in MIMIC-IV cohort (A, C) and eICU-CRD cohort (B, D)

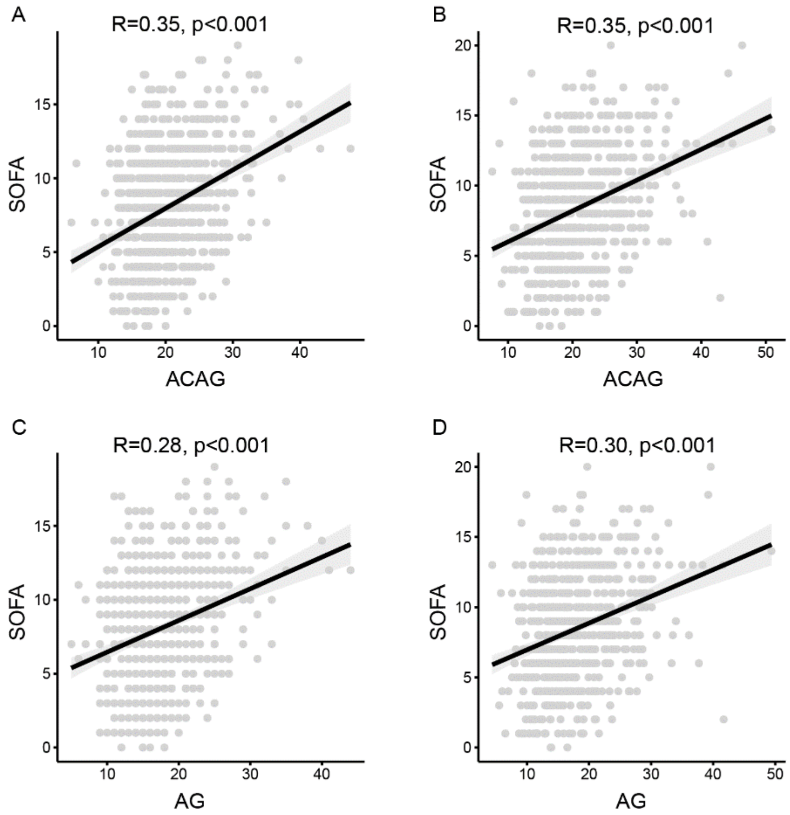

eFigure3: Kaplan–Meier survival curve of ACAG levels and 28-day all-cause mortality

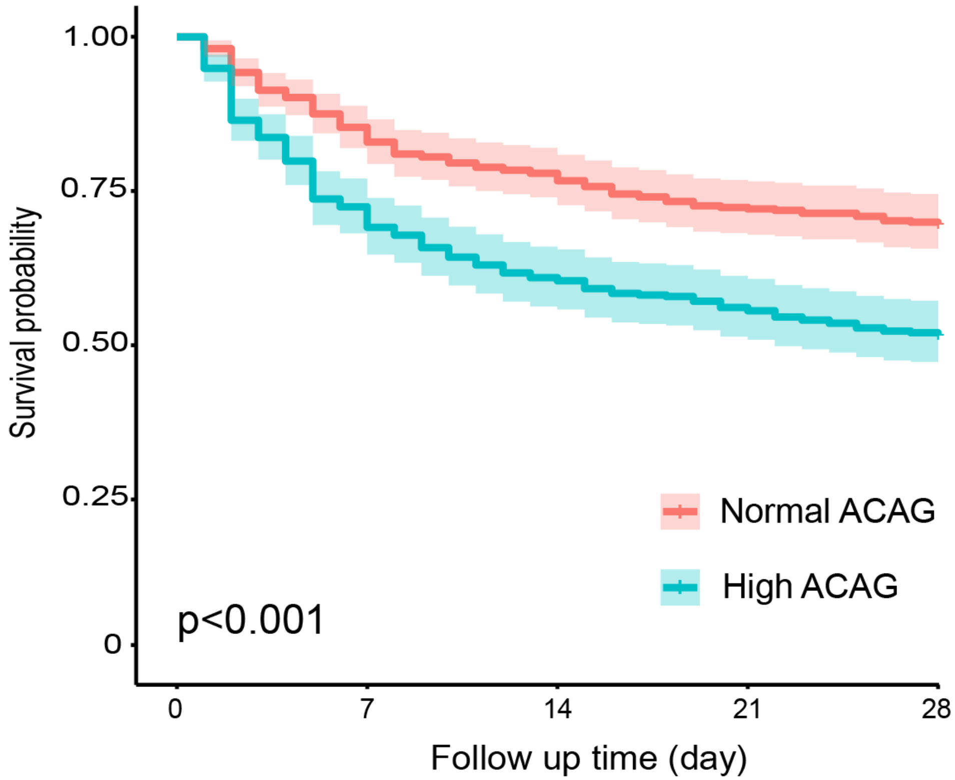

eFigure4: Cumulative incidence ratio of earlier discharge alive in the ICU in MIMIC-IV cohort (A) and eICU-CRD cohort (B)

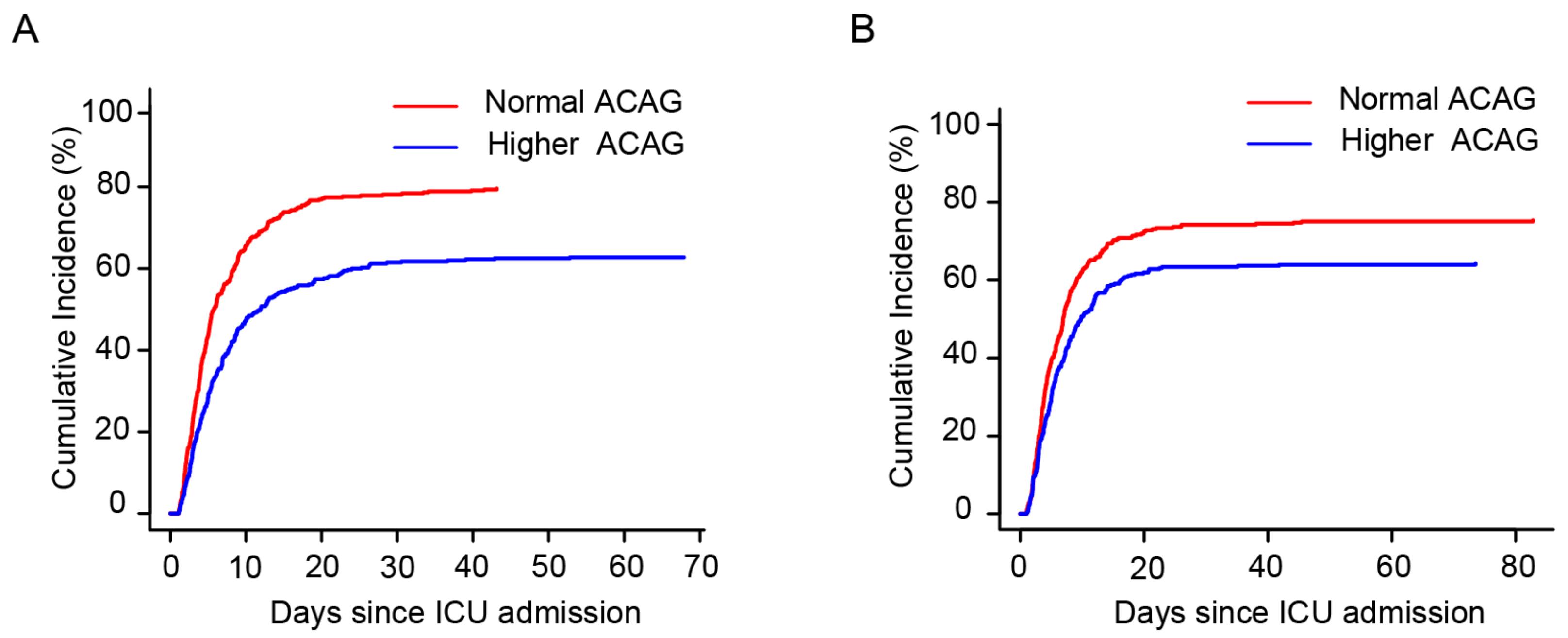

eFigure5: Subgroup analysis

ACAG: albumin corrected anion gap, HR: hazard ratio, CI: confidence interval, AMI: acute myocardial infarction, AF: atrial fibrillation, COPD: chronic obstructive pulmonary disease, AKI: acute kidney injury, SOFA: Sequential Organ Failure Assessment

Subgroup	ACAG<20	ACAG≥20	HR (95% CI)	p for interaction
Age(years)				0.830
Age<65	143	138	1.35 (0.82 to 2.22)	
Age≥65	273	254	1.43 (1.09 to 1.89)	
Gender				0.923
Female	188	159	1.41 (0.99 to 2.00)	
Male	228	233	1.44 (1.03 to 2.01)	
AMI				0.131
No	229	230	1.22 (0.89 to 1.68)	
Yes	187	162	1.75 (1.21 to 2.53)	
Cardiomypathy				0.586
No	311	291	1.47 (1.11 to 1.95)	
Yes	105	101	1.26 (0.77 to 2.07)	
AF				0.494
No	217	198	1.31 (0.93 to 1.85)	
Yes	199	194	1.54 (1.10 to 2.16)	
Valvular disorders				0.660
No	266	249	1.48 (1.10 to 1.99)	
Yes	150	143	1.32 (0.87 to 2.00)	
COPD				0.943
No	375	362	1.42 (1.09 to 1.85)	
Yes	41	30	1.46 (0.67 to 3.20)	
Diabetes				0.260
No	300	225	1.58 (1.16 to 2.16)	
Yes	116	167	1.20 (0.82 to 1.76)	
AKI				0.400
No	158	81	1.16 (0.68 to 1.99)	
Yes	258	311	1.50 (1.13 to 1.98)	
Hypoalbuminemia				0.157
No	221	251	1.24 (0.92 to 1.67)	
Yes	195	141	1.76 (1.16 to 2.67)	
SOFA				0.182
<8	221	138	1.78 (1.18 to 2.68)	
≥8	195	254	1.27 (0.94 to 1.71)	

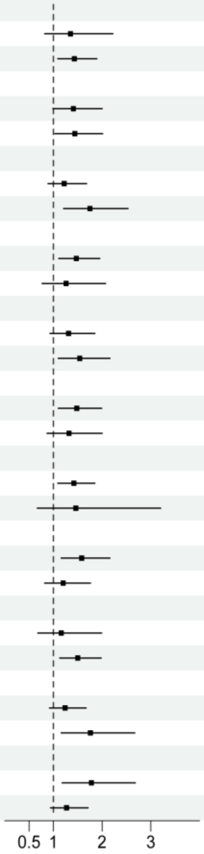

Supplement: online supplemental file 1 [file bmjopen-14-10-s001.pdf]
