# Supplementary material for: Relationship between the albumin-corrected anion gap and short-term prognosis among patients with cardiogenic shock: a retrospective analysis of the MIMIC-IV and eICU databases
Source: BMJ Open. 2024 Oct 2;14(10):e081597. doi: 10.1136/bmjopen-2023-081597 (PMC11448225; doi:10.1136/bmjopen-2023-081597)
Supplement: Supplementary file 2 [file bmjopen-2023-081597.reviewer_comments.pdf]

## PEER REVIEW HISTORY

BMJ Open publishes all reviews undertaken for accepted manuscripts. Reviewers are asked to complete a checklist review form and are provided with free text boxes to elaborate on their assessment. These free text comments are reproduced below.

## ARTICLE DETAILS

| | |
|---|---|
| **TITLE (PROVISIONAL)** | Relationship between the albumin-corrected anion gap and short-term prognosis among patients with cardiogenic shock: a retrospective analysis of the MIMIC-IV and eICU databases |
| **AUTHORS** | Wang, Yuxing; Tao, Yuhang; Yuan, Ming; Yu, Pengcheng; Zhang, Kai; Ying, Hangying; Jiang, Ruhong |

## VERSION 1 - REVIEW

| | |
|---|---|
| **REVIEWER NAME** | Kechida, Melek |
| **REVIEWER AFFILIATION** | Fattouma Bourguiba University Hospital of Monastir, Internal Medicine and Endocrinology Department |
| **REVIEWER CONFLICT OF INTEREST** | no competing interests |
| **DATE REVIEW RETURNED** | 21-Dec-2023 |

| | |
|---|---|
| **GENERAL COMMENTS** | The authors reported a multi-center, retrospective, observational study with a primary endpoint the short-term all-cause mortality including ICU, in-hospital, and 28-day mortality, and as secondary endpoints, 28-day free from ICU and length of time intensive care needed. The overall study is well-written, methods section is well-detailed. The results are well discussed. The manuscript could be accepted with no changes |

| | |
|---|---|
| **REVIEWER NAME** | Meynaar, IA |
| **REVIEWER AFFILIATION** | Intensive Care Unit, Reinier de Graaf Hospital |
| **REVIEWER CONFLICT OF INTEREST** | None |
| **DATE REVIEW RETURNED** | 22-Dec-2023 |

| | |
|---|---|
| **GENERAL COMMENTS** | Wang et al studied the association between mortality and anion gap AG and albumin corrected anion gap ACAP in two combined retrospective cohorts of patients with cardiogenic shock treated in the ICU to find that both were associated with mortality, but that AGAP was a stronger predictor of mortality than plain AG. The authors used extensive statistical methods underlining to the robustness of their findings. The manuscript is written clearly and precisely. Strengths and weaknesses are described adequately.<br><br>I have only a few comments:<br><br>Pg 4 line 21-22: how was cardiogenic shock defined? |

| | |
|---|---|
| | The manuscript is complete but also rather long and extensive. The discussion for instance could be shortened by removing the arguments that are already made in the introduction. |

| | |
|---|---|
| **REVIEWER NAME** | Berezin, A. E. |
| **REVIEWER AFFILIATION** | State Med Univ |
| **REVIEWER CONFLICT OF INTEREST** | I have no competing interests |
| **DATE REVIEW RETURNED** | 22-Dec-2023 |

| | |
|---|---|
| **GENERAL COMMENTS** | The authors submitted a research article in which they investigated the association between albumin corrected anion gap and the prognosis of cardiogenic shock. They included 808 and 700 individuals diagnosed with CS from edical Information Mart for Intensive Care and eICU Collaborative Research Database respectively and found that patients with a higher albumin corrected anion gap level exhibited increased short-term all cause, ICU, in-hospital mortalities. Finally, they established that albumin corrected anion gap value was independently and strongly associated with the prognosis of cardiogenic shock, which was superior than the conventional anion gap rate. Although the findings are intriguing, I would like to make several comments.<br>1. It remained unclear what SCAI CS stages had the patients. Please, check and interpretad widely.<br>2. Did the authors adjust co-factors to acute renal failure ?<br>3. Whether actual medications were found as plausible co-factors for regression ?<br>4. 43% patients were found to be post-AMI. Whether post-PCI TIMI was detected to be further interpreted or revascularization was incompleted ? Please, give thorough explanation of this issue. |

| | |
|---|---|
| **REVIEWER NAME** | Nochioka, Kotaro |
| **REVIEWER AFFILIATION** | Tohoku University Graduate School of medicine, Cardiovascular Medicine |
| **REVIEWER CONFLICT OF INTEREST** | Nothing to declare. |
| **DATE REVIEW RETURNED** | 27-Dec-2023 |

| | |
|---|---|
| **GENERAL COMMENTS** | This study is interesting to evaluate the prognostic significance of albumin-corrected anion gap (ACAG) in patients after cardiogenic shock. I have a few comments to the authors.<br><br>1. There are several risk scores (Journal of the American Heart Association. 2023;12:e029232, Front Cardiovasc Med. 2022; 9: 842056. etc..) Is ACAG superior to these risk scores? Does ACAG have addictive value for risk prediction?<br><br>2. Overall the AUCs of ACAG are relatively low: 0.59 to 0.65.<br><br>3. Figure 2. What is the meaning of P for non-linear? If P for non-linear is <0.05, the curve should be linear or non-linear?<br><br>4. Are there differences in clinical characteristics between included patients and excluded in MIMIC-IV and eICU-CRD cohorts? A |

| | |
|---|---|
| | supplemental table would be nice to see any difference in included patients (n=808) and excluded (n=1684-808) in MIMIC-IV, and included (n=700) and excluded (n=1289-700) in eICU-CRD. |

**VERSION 1 – AUTHOR RESPONSE**

Reviewer: 1

Dr. Melek Kechida, Fattouma Bourguiba University Hospital of Monastir, University of Monastir

Comments to the Author:

The authors reported a multi-center, retrospective, observational study with a primary endpoint the short-term all-cause mortality including ICU, in-hospital, and 28-day mortality, and as secondary endpoints, 28-day free from ICU and length of time intensive care needed. The overall study is well-written, methods section is well-detailed. The results are well discussed. The manuscript could be accepted with no changes

Response

Thank you for your thorough review and positive feedback on our manuscript. We are pleased to hear that you found the paper well-written, with a detailed methods section and well-discussed results. Your recommendation for acceptance without changes is highly encouraging. We will prepare the necessary documents for publication in accordance with the journal's further requirements and ensure the manuscript maintains a high standard of quality.

Reviewer: 2

Dr. IA Meynaar, Intensive Care Unit, Reinier de Graaf Hospital

Comments to the Author:

Wang et al studied the association between mortality and anion gap AG and albumin corrected anion gap ACAP in two combined retrospective cohorts of patients with cardiogenic shock treated in the ICU to find that both were associated with mortality, but that AGAP was a stronger predictor of mortality than plain AG. The authors used extensive statistical methods underlining to the robustness of their findings. The manuscript is written clearly and precisely. Strengths and weaknesses are described adequately.

I have only a few comments:

Pg 4 line 21-22: how was cardiogenic shock defined?

The manuscript is complete but also rather long and extensive. The discussion for instance could be shortened by removing the arguments that are already made in the introduction.

Response

Thank you for your thoughtful review and the positive remarks about the clarity and precision of our manuscript. Your feedback is greatly appreciated and will be used to improve our paper.

Comments

Regarding the definition of cardiogenic shock (CS), in our study, CS was defined based on the diagnostic codes from the MIMIC-IV and eICU-CRD databases. These codes are in accordance with the standard clinical definitions. We will clarify this definition in the revised version of the manuscript.

Reviewer: 3

Dr. A. E. Berezin, State Med Univ

Comments to the Author:

The authors submitted a research article in which they investigated the association between albumin corrected anion gap and the prognosis of cardiogenic shock. They included 808 and 700 individuals diagnosed with CS from edical Information Mart for Intensive Care and eICU Collaborative Research Database respectively and found that patients with a higher albumin corrected anion gap level exhibited increased short-term all cause, ICU, in-hospital mortalities. Finally, they established that albumin corrected anion gap value was independently and strongly associated with the prognosis of cardiogenic shock, which was superior than the conventional anion gap rate. Although the findings are intriguing, I would like to make several comments.

1. It remained unclear what SCAI CS stages had the patients. Please, check and interpretad widely.

2. Did the authors adjust co-factors to acute renal failure ?

3. Whether actual medications were found as plausible co-factors for regression ?

4. 43% patients were found to be post-AMI. Whether post-PCI TIMI was detected to be further interpreted or revascularization was incompleted ? Please, give thorough explanation of this issue.

Response

Thank you for your insightful comments and for recognizing the potential significance of our findings. We appreciate the opportunity to clarify and expand upon the points you have raised.

Comment_1:

Regarding the SCAI CS stages of the patients, we regret that due to the retrospective nature of our study and specific data limitations, particularly the absence of critical parameters like lactate levels and pH values in the Medical Information Mart for Intensive Care (MIMIC-IV) and eICU Collaborative Research Database, we were unable to calculate the SCAI CS stages accurately. This limitation has been acknowledged in the limitations section of our manuscript. We recommend that future prospective studies include these essential parameters to facilitate precise SCAI CS staging and assess its impact on patient outcomes more accurately.

Comment_2:

Due to the variability in diagnostic information between the two databases used, we adjusted for AKI in the Medical Information Mart for Intensive Care (MIMIC-IV) and for acute renal failure in the eICU Collaborative Research Database (eICU-CRD)

Comment_3:

In response to your third comment about the role of actual medications, specifically vasoactive medications such as norepinephrine, epinephrine, dopamine, and dobutamine, as potential co-factors in our study, I want to clarify our analytical approach. While these medications are indeed related to clinical outcomes in cardiogenic shock, we assessed their potential confounding effect on the relationship between albumin corrected anion gap and the prognosis of cardiogenic shock using the 'change in estimate' method. This method evaluates how much the inclusion of a potential confounder changes the effect size of the primary exposure-outcome relationship. Our findings showed that incorporating these vasopressors as co-factors in the regression model altered the effect estimates by less than 10%. Following established epidemiological practices, a variable that changes the effect estimate by less than 10% is generally not considered a significant confounder. Therefore, based on this criterion, we decided not to include these vasopressor medications in the final model, as their impact on the effect size was minimal.

Comment_4:

Given the retrospective nature of our research, which relied on data extracted from the Medical Information Mart for Intensive Care (MIMIC-IV) and eICU Collaborative Research Database, detailed procedural outcomes such as post-PCI TIMI flow grades and the completeness of revascularization are not consistently available or documented. Therefore, despite the relevance of these factors to the prognosis of cardiogenic shock, our study's retrospective design and the limitations of the existing database records precluded a comprehensive analysis of these aspects. We acknowledge this as a limitation and suggest that further studies with detailed angiographic data could provide more insights into this aspect.

Reviewer: 4

Dr. Kotaro Nochioka, Tohoku University Graduate School of medicine

Comments to the Author:

This study is interesting to evaluate the prognostic significance of albumin-corrected anion gap (ACAG) in patients after cardiogenic shock. I have a few comments to the authors.

1. There are several risk scores (Journal of the American Heart Association. 2023;12:e029232, Front Cardiovasc Med. 2022; 9: 842056. etc..) Is ACAG superior to these risk scores? Does ACAG have addictive value for risk prediction?

2. Overall the AUCs of ACAG are relatively low: 0.59 to 0.65.

3. Figure 2. What is the meaning of P for non-linear? If P for non-linear is <0.05, the curve should be linear or non-linear?

4. Are there differences in clinical characteristics between included patients and excluded in MIMIC-IV and eICU-CRD cohorts? A supplemental table would be nice to see any difference in included patients (n=808) and excluded (n=1684-808) in MIMIC-IV, and included (n=700) and excluded (n=1289-700) in eICU-CRD.

Response

Thank you for your insightful comments and for acknowledging the interest in our study regarding the prognostic significance of the albumin-corrected anion gap (ACAG) in patients after cardiogenic shock. We address your comments as follows:

Comment_1:

Concerning the BOS,MA2 score referenced in Journal of the American Heart Association. 2023;12:e029232, we performed rigorous statistical analyses. Our results indicated negative outcomes, suggesting that ACAG, in both cohorts, did not significantly outperform the BOS,MA2 score in predicting prognosis. Then, we integrated the albumin-corrected anion gap (ACAG) into the prognostic scoring model, attributing one point for an ACAG value of 20 or higher, thereby replacing the traditional anion gap (AG) metric. Despite this adjustment, the results revealed that BOS,MA2 score (ACAG) did not significantly improve the predictive efficiency of the original BOS,MA2 score (AG) for prognosis prediction.

Concerning the CSP score referenced in Front Cardiovasc Med (2022; 9: 842056), we acknowledge the constraints in our study posed by the unavailability of data on ejection fraction and cardiopulmonary resuscitation duration. We were unable to perform CSP scoring due to these data gaps stemming from the retrospective nature of our database study

Comment_2:

Concerning the AUCs of ACAG, we acknowledge that the ranges of 0.59 to 0.65 indicate moderate predictive power. However, it is crucial to highlight our findings regarding the comparative effectiveness of ACAG over the traditional anion gap (AG).

Comment_3:

For Figure 2 and the interpretation of the P-value for non-linearity, this statistical measure was intended to test whether the relationship between ACAG and mortality follows a linear or non-linear trajectory. A P-value <0.05 suggests that the relationship is non-linear. We will clarify this point and ensure the figure and its caption accurately reflect the statistical analysis.

Comment_4:

Regarding the differences in clinical characteristics between included and excluded patients, we recognize the importance of this analysis to understand potential selection bias. We will provide a supplemental table in the revised manuscript, detailing the clinical characteristics of both included and excluded patients in the MIMIC-IV and eICU-CRD cohorts, to offer a comprehensive view of the study population.

**VERSION 2 - REVIEW**

| | |
|---|---|
| **REVIEWER NAME** | Berezin, A. E. |
| **REVIEWER AFFILIATION** | State Med Univ |
| **REVIEWER CONFLICT OF INTEREST** | None |
| **DATE REVIEW RETURNED** | 10-Jun-2024 |

| | |
|---|---|
| **GENERAL COMMENTS** | The authors submitted the critically revised version of the paper along with clear reply to the editor and reviewers. I have no serious consens about the article in its revised version. |

| | |
|---|---|
| **REVIEWER NAME** | Nochioka, Kotaro |
| **REVIEWER AFFILIATION** | Tohoku University Graduate School of medicine, Cardiovascular Medicine |
| **REVIEWER CONFLICT OF INTEREST** | There is nothing to declare. |
| **DATE REVIEW RETURNED** | 14-Jun-2024 |

| | |
|---|---|
| **GENERAL COMMENTS** | The authors addressed my comments adequately. I do not have further comments at this point. |
